# Effects of nutrient level and planting density on population relationship in soybean and wheat intercropping populations

**DOI:** 10.1371/journal.pone.0225810

**Published:** 2019-12-02

**Authors:** Jialing Huang, Yihang Li, Yu Shi, Lihong Wang, Qing Zhou, Xiaohua Huang

**Affiliations:** 1 State Key Laboratory of Food Science and Technology, School of Environment and Civil Engineering, Jiangsu Key Laboratory of Anaerobic Biotechnology, Jiangnan University, Wuxi, China; 2 Jiangsu Cooperative Innovation Center of Water Treatment Technology and Materials, Suzhou University of Science and Technology, Suzhou, China; 3 Jiangsu Collaborative Innovation Center of Biomedical Functional Materials, Jiangsu Key Laboratory of Biomedical Materials, School of Chemistry and Materials Science, Nanjing Normal University, Nanjing, China; Northwest Agriculture and Forestry University, CHINA

## Abstract

A positive interaction between plant populations is a type of population relationship formed during long-term evolution. This interaction can alleviate population competition, improve resource utilization in populations, and promote population harmony and community stability. However, cultivated plant populations may have insufficient time to establish a positive interaction, thereby hindering the formation of the positive interaction. As current studies have not fully addressed these issues, our study established soybean/wheat intercropping populations beneficial for growth and explored the effects of nutrient level and planting density on the positive interaction between the two crops. Changes across population modules in both sole cropping and intercropping populations of soybean and wheat were analyzed. Results using nutrient levels of ½- or ¼-strength Hoagland solution indicated that soybean/wheat intercropping population modules significantly increased at low planting densities (D_20_ and D_26_) and significantly decreased at high planting densities (D_32_ and D_60_). Therefore, as planting density increased, the modules of both intercropping populations initially increased before decreasing. Similarly, positive interaction initially strengthened before weakening. Moreover, at an intermediate planting density, the population modules reached their maxima, and the positive interaction was the strongest. Under the same planting density, ¼-strength Hoagland solution recorded better growth for the soybean/wheat intercropping population modules compared to results using the ½-strength Hoagland solution. These findings indicated that low nutrient level can increase the positive interaction of intercropping populations at a given planting density, and that environmental nutrient level and population planting densities constrain the positive interaction between soybean and wheat populations in the intercropping system. This study highlights issues that need to be addressed when constructing intercropping populations.

## Introduction

In nature, populations form the basic unit of existence, reproduction and species evolution [1]; species present today are the result of evolution and preservation of populations over millions of years. Interspecific relationships are extremely complex, and they can be roughly divided into negative, neutral and positive interactions [2]. Different population relationships affect both the utilization and efficiency of resources available for the species [3, 4]. Positive interaction, a type of population relationship formed during population evolution, has been suggested to avoid population competition, improve resource utilization efficiency, promote harmonious relationships, stabilize communities, and improve biodiversity [5, 6].

The emergence of positive interaction between populations is a sign of ecosystem maturity. The basis of positive interaction is the divergence of resource utilization modes between populations, reflected by the adaptation of the population module [7] and its function in the environment. For example, in soybean/wheat intercropping populations, soybean plants have straight root systems and broad leaves which can utilize nutrients and water in deeper soil layers. In contrast, wheat plants have fibrous roots and narrow leaves, suitable for effectively utilizing nutrients and water in the upper soil layers. Consequently, in soybean/wheat intercropping populations, roots from both plants absorb and utilize the greatest amount of nutrients and water [8]. Furthermore, their leaves fully utilize the aboveground space and illumination. For natural populations, the positive interaction between populations is the result of long-term environmental selection and adaptation. In an artificial environment, such as an agricultural plantation, long-term natural selection has either not occurred, or the time to establish positive interaction between populations may not be sufficient. In such a system, it is unknown whether the positive interaction between intercropping populations is stable or weak. In other words, constraints (if any) on positive interactions between intercropping populations remains unknown. It is also unknown if changes to environmental nutrient level and population planting density affect positive interaction on intercropping populations.

To investigate these issues, intercropping populations of soybean/wheat (mutually beneficial crops) and their positive interaction were examined. Changes in the positive interaction relationship between soybean/wheat intercropping populations were determined using both the aboveground and belowground modules. In addition, an experimental design using two nutrient levels, four population densities and three planting methods was adopted to investigate the effects of both factors on the positive interaction of soybean/wheat intercropping populations. Our results provide a reference for a further understanding of the positive interaction between mutually beneficial populations, an understanding that is important to improve utilization of environmental resources and crop productivity.

## Materials and methods

### Crop culture and treatment

In consideration of the different life forms of soybean and wheat, and to alleviate excessive shading of wheat seedlings by established soybean plants, we initially planted wheat seedlings before soybeans were planted. Wheat seeds (‘BainongAikang 58’ cultivar, Wuxi Seed Co., Ltd., China) used in our study were disinfected with 0.1% HgCl_2_ for 10 min before being rinsed three times with deionized water. The seeds were then soaked in deionized water for 24 h, placed in a Petri dish with three layers of wet filter paper, germinated in a constant temperature incubator (25 ± 1.0°C), and replenished with water three times a day. When the height of the wheat seedlings were 6–7 cm, the germinated wheat plants were transplanted into plastic pots (290 × 290 × 250 mm) and watered with deionized water in a greenhouse (temperature, 25°C; light intensity, 300 μmol m^-1^ s^-1^; photoperiod, 16 h/8 h) [9]. Sponge and foam boards (290 × 290 × 15 mm) with uniform holes (diameter = 2 cm) were used to predefine the spacing of the wheat plants (1:1 interval planting for soybean and wheat). Soybean seeds (‘Zhonghuang 25’ cultivar, Wuxi Seed Co., Ltd., China) were germinated under the same methods and conditions used for wheat. When the soybean radicles had grown to 2 cm, the seedlings were transferred into the remaining holes (diameter = 1 cm) and they were intercropped with the established wheat plants (planted in 1:1 intervals) [10, 11]. Control samples were established using sole cropping populations with the same density. Plant densities in sole cropping and intercropping populations were set at 20, 26, 32 and 60 plants pot^-1^ [12, 13], expressed as D_20_, D_26_, D_32_ and D_60_, respectively. When the first true soybean leaf unfolded, each of the two treatment groups were cultured with ½-strength (pH 7.0) [14] and ¼-strength Hoagland nutrient solution (pH 7.0) [15], respectively. The control group used to analyze nutrient levels consisted of soybean and wheat seedlings cultured in ½-strength Hoagland solution; the low-level nutrient group consisted of seedlings cultured in ¼-strength Hoagland solution. In total, 24 treatment groups were used in our experiment (two nutrient levels × four planting densities × three planting ways). All treatments included an aeration period of three min every three hours to stabilize the pH value of the nutrition solutions. After treatment for 20 days, experimental indices were measured. For each treatment, five pots containing soybean and wheat populations were randomly selected as experimental samples. All assays were repeated in triplicate.

### Determination of aboveground and belowground population modules

Leaf area for both soybean and wheat populations were measured using a CI-203 laser leaf area meter (CID, Inc., Camas, WA, USA) [16]. Stem diameter and plant height were measured using a Vernier caliper and ruler, respectively. Fresh roots from both plant species were collected and washed three times with distilled water to ensure they were clean. A root automatism scan apparatus (Perfection V700 Photo, Seiko Epson Corp., Suwa, Japan) equipped with WinRHIZO software (version 2009a, Regent Instruments, Quebec City, Quebec, Canada) was used to determine root phenotypes [17]. Root segments were placed on the scanning apparatus in a transparent plastic tray filled with deionized water. WinRHIZO 2009a software was used to evaluate the following root module phenotype parameters: root tip number, total root length, root volume and root surface area [18]. The dry weights of leaves, stems and roots from both populations were determined after drying at 80°C until a constant weight was recorded [19].

### Statistical analysis

Ten important module indices were selected as candidate indices to assess the positive interaction between soybean and wheat populations. These indices were used as the basis for performing principal component analysis (PCA). After standardizing the original data, a specific number of principal components were extracted and the principal component values were calculated according to the principle of accumulated variance contribution rate > 70%. Finally, PCA scores were calculated by integrating principal components [20]. This analysis was undertaken using SPSS version 17.0 (SPSS Inc., Chicago, IL, USA). Following analysis of variance (ANOVA), Fisher’s least significant difference (LSD) test was undertaken to determine the significance of the differences among treatments (*p* < 0.05). All figures were drawn using Origin 8.5 (Originlab, Northampton, MA, USA).

## Results

### Effects of nutrient level and planting density on aboveground modules

**Fig 1:**
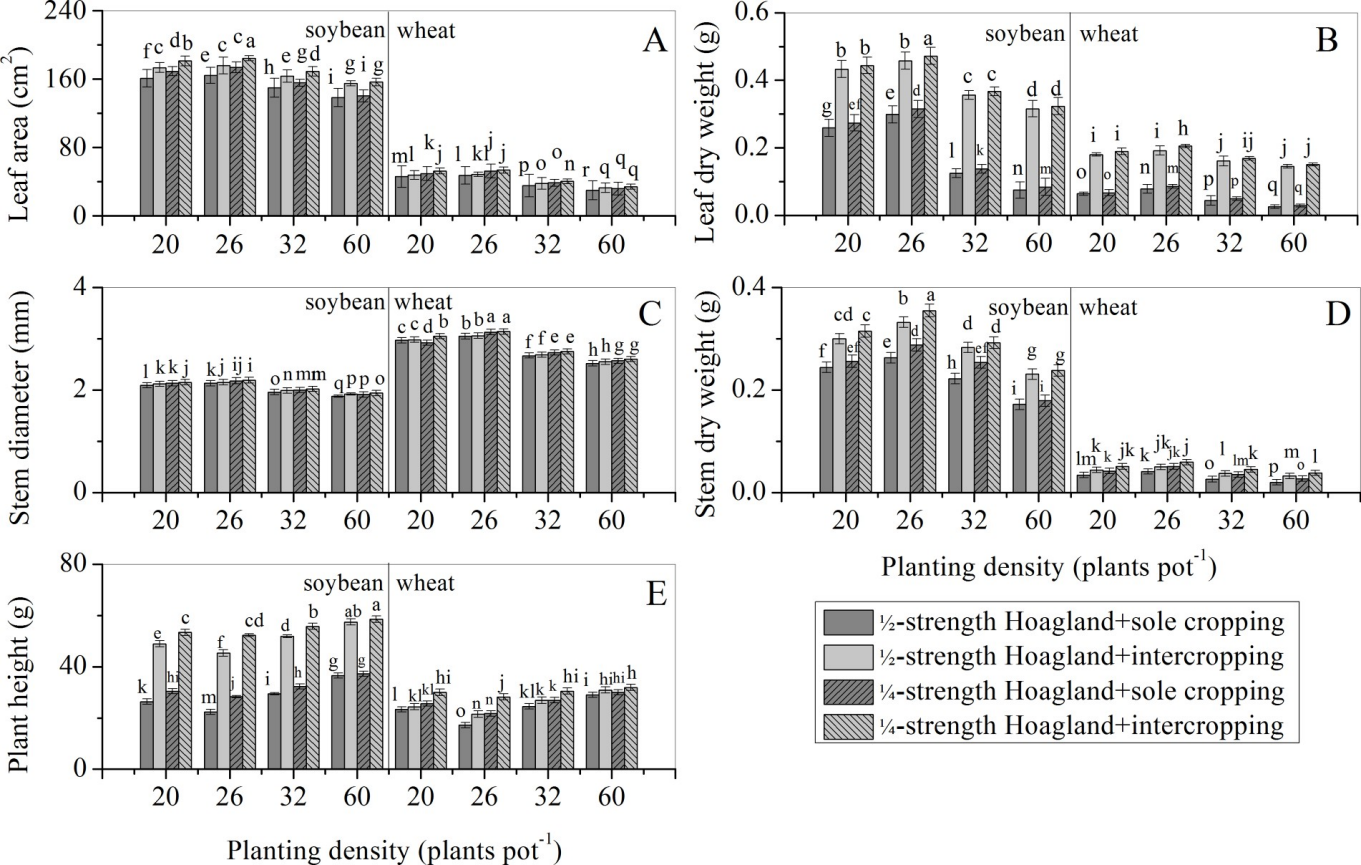
Effects of nutrient level and planting density on aboveground modules of soybean/wheat sole cropping and intercropping populations. Data are expressed as means ± standard errors of five replicates, and the error bars represent standard errors. Treatments marked with the same letter are not significantly different according to Fisher’s LSD test (*p* < 0.05).

Fig 1 shows, for both nutrient levels, the leaf area, leaf dry weight, stem diameter, and stem dry weight of both soybean and wheat sole cropping populations initially increased before decreasing, while plant height recorded an initial decrease before increasing as planting density increased. These effects were strongest at D_26_, followed by D_20_, D_32_, and D_60_ in descending order. Importantly, the changing rule of these indices for both intercropping populations was consistent with their sole cropping populations. The modules of intercropping populations were larger than those of sole cropping populations, and the extent of change of the modules were smaller than those of the sole cropping populations. This result indicated that soybean/wheat intercropping populations formed mutually beneficial populations. Our results also indicated that D_26_, recording a stronger positive interaction compared to other planting densities (D_20_, D_32_, and D_60_), was a suitable planting density for soybean/wheat intercropping populations. In addition, as planting density increased in both populations, the restriction of nutrient level on population modules increased and the positive interaction between the two populations weakened. When the planting density of both populations remained constant, the module indices under the ¼-strength Hoagland treatment were higher than those under the ½-strength Hoagland treatment. This result indicated that low nutrient condition promoted positive interaction between populations.

### Effects of nutrient level and planting density on belowground modules

**Fig 2:**
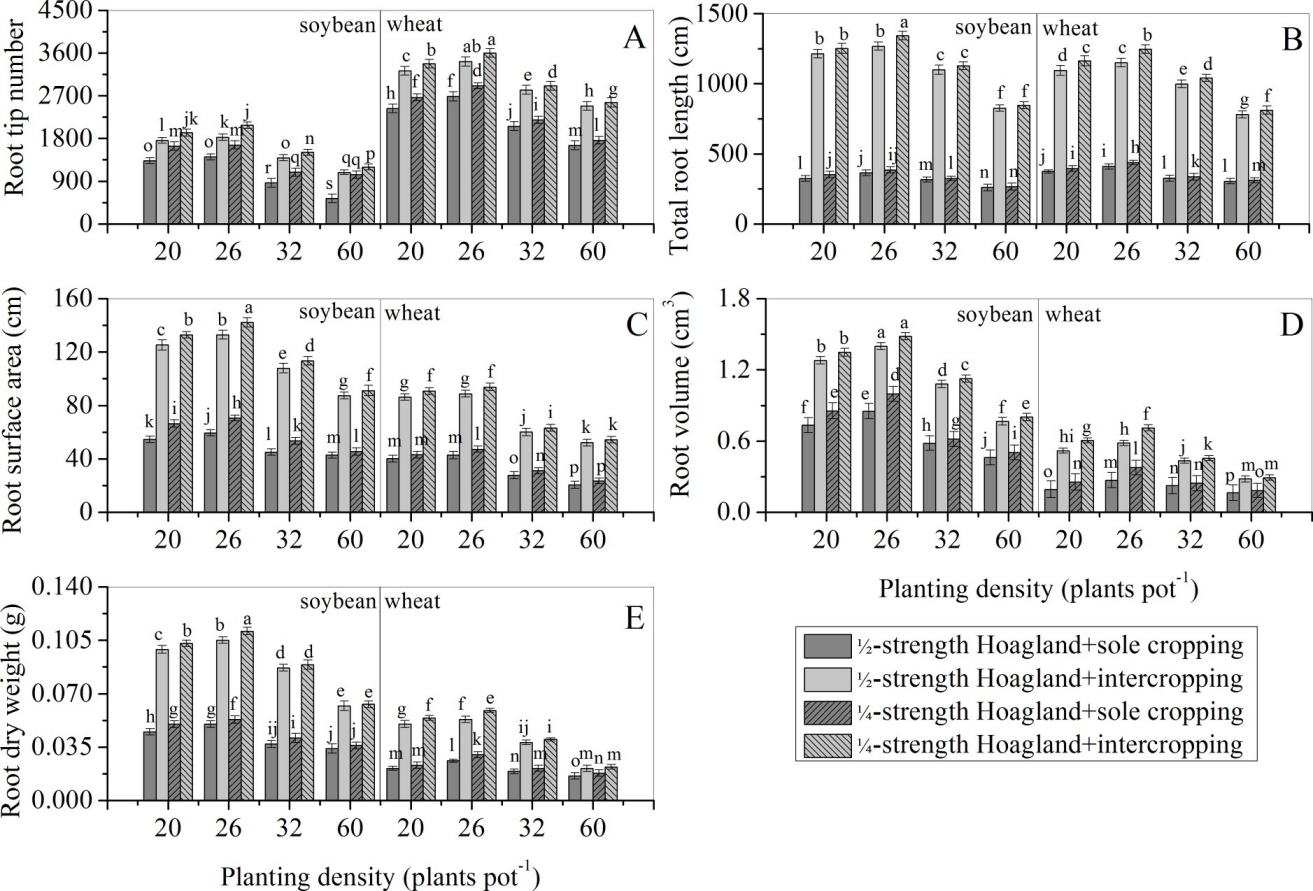
Effects of nutrient level and planting density on underground modules of soybean/wheat sole cropping and intercropping populations. Data are expressed as means ± standard errors of five replicates, and the error bars represent standard errors. Treatments marked with the same letter are not significantly different according to Fisher’s LSD test (*p* < 0.05).

**Fig 3:**
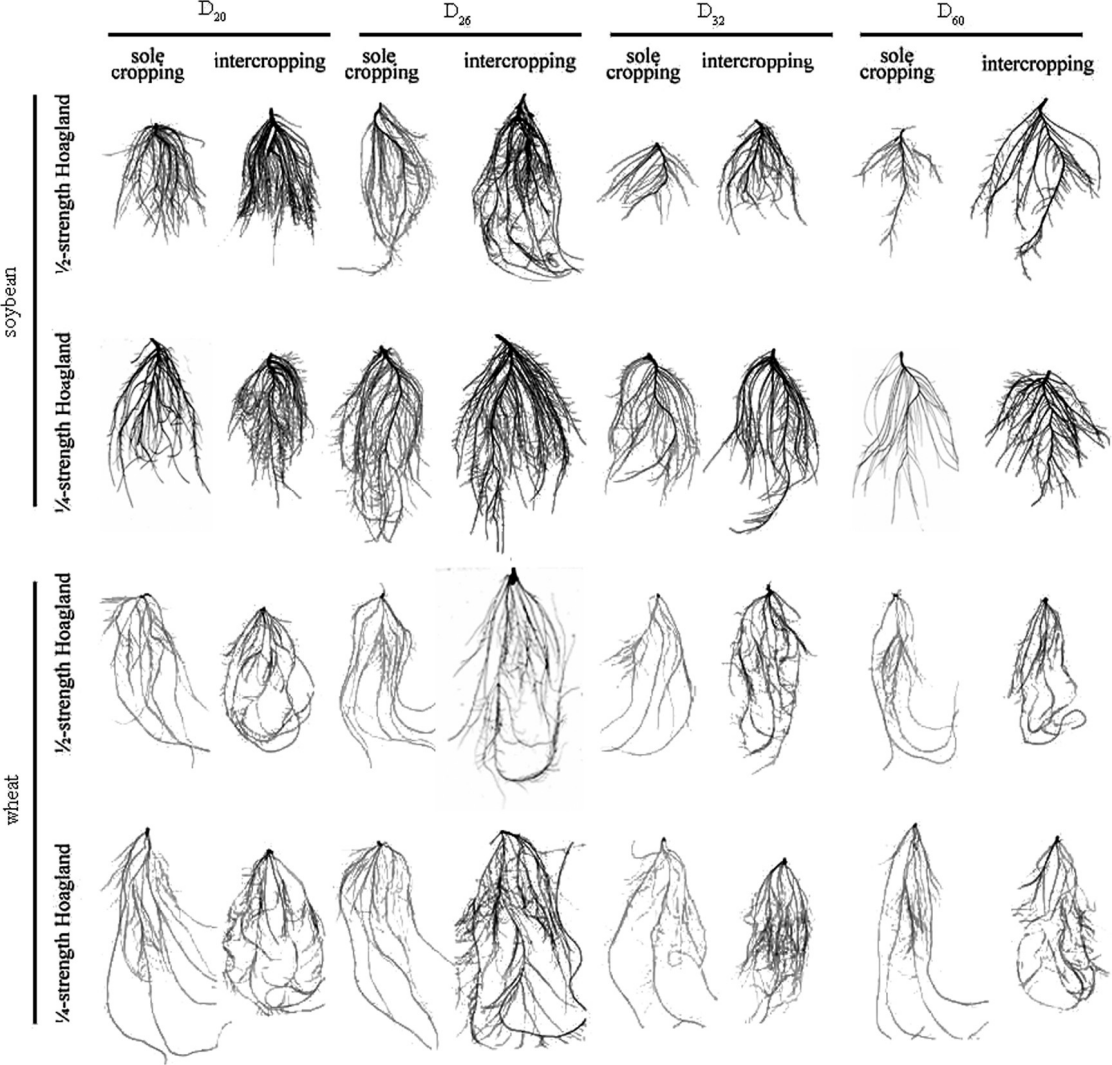
Root images for soybean/wheat sole cropping and intercropping populations under different nutrient levels and planting densities.

Figs 2 and 3 show, for both nutrient levels, the root tip number, root length, root surface area, root volume, and root dry weight of both soybean and wheat sole cropping populations initially increased before decreasing with an increase in planting density. These effects were strongest at D_26_, followed by D_20_, D_32_, and D_60_ in descending order. Interestingly, the changing rule of the above indices of soybean and wheat intercropping populations was consistent with their sole cropping populations. The modules of intercropping populations were greater than those of sole cropping populations and the extent of change of the modules were smaller than those of the sole cropping populations. This result indicated that soybean/wheat intercropping populations were mutually beneficial populations. As D_26_ recorded a stronger positive interaction compared to other planting densities (D_20_, D_32_, and D_60_), these results indicated a suitable planting density for soybean/wheat intercropping populations. In addition, with increasing planting density of both populations, restrictions on population modules imposed by nutrient levels increased and the positive interaction between both populations weakened. When the planting density of populations remained constant, the module indices under the ¼-strength Hoagland treatment were higher than those under the ½-strength treatment, indicating that low nutrient condition promoted positive interaction between populations.

### PCA of the positive interaction between soybean and wheat intercropping populations

The morphology and relationship of plant populations are moldable and are reflected by the adaptive changes of population module indices [21, 22]. As an evaluation of population relationships is not accurate solely based on one or several module indices, we therefore used PCA to comprehensively evaluate the relationship between soybean and wheat populations. This method resulted in simple, scientific and reasonable outcomes by introducing multiple indices and summarizing the indices into several principal components through dimensionality reduction [23–25]. The PCA process used in this study was: (1) Indices normalization—raw data of module indices in all treatment groups were standardized to establish a comparable basis of PCA (Tables 1 and 2). (2) Based on data in Tables 1 and 2, the multiple indices were summarized into two principal components (Table 3). As the cumulative contribution rate of the first two principal components (93.051%) was significantly greater than 70%, the first two principal components were extracted and two principal component load matrices (U1 and U2) were calculated. (3) Normalized data from Tables 1 and 2 and load matrices U1 and U2 from Table 3 were summed to receive a PCA score formula (Table 4):

PCAscore=(67.827×PC1+25.224×PC2)/93.051 (1)

**Table 1:** Standardized data for population modules in soybean/wheat sole cropping and intercropping populations at normal nutrient level and different planting densities.

**Planting density**	**Planting mode**	**Crop type**	^a^ (Z Leaf area)	^a^ (Z Leaf dry weight)	^a^ (Z Stem diameter)	^a^ (Z Stem dry weight)	^a^ (Z Plant height)	^a^ (Z Root tip number)	^a^ (Z Total root length)	^a^ (Z Root surface area)	^a^ (Z Root volume)	^a^ (Z Root dry weight)
D_20_	sole cropping	soybean	0.931	0.390	-0.807	0.772	-0.640	-0.864	-0.980	-0.450	0.225	-0.152
		wheat	-0.913	-1.030	1.212	-0.985	-0.888	0.486	-0.852	-0.874	-1.173	-1.007
	intercropping	soybean	1.133	1.658	-0.739	1.240	1.241	-0.341	1.272	1.629	1.641	1.774
		wheat	-0.884	-0.185	1.235	-0.901	-0.804	1.461	0.967	0.481	-0.329	0.027
D_26_	sole cropping	soybean	0.988	0.682	-0.716	0.930	-0.975	-0.765	-0.876	-0.307	0.530	0.027
		wheat	-0.888	-0.928	1.395	-0.926	-1.407	0.802	-0.763	-0.795	-0.973	-0.829
	intercropping	soybean	1.173	1.840	-0.670	1.508	0.940	-0.253	1.408	1.850	1.951	1.988
		wheat	-0.870	-0.098	1.418	-0.851	-1.046	1.701	1.109	0.554	-0.163	0.134
D_32_	sole cropping	soybean	0.754	-0.586	-1.106	0.588	-0.386	-1.437	-0.997	-0.730	-0.166	-0.437
		wheat	-1.079	-1.176	0.523	-1.052	-0.794	0.032	-0.973	-1.238	-1.090	-1.079
	intercropping	soybean	0.974	1.097	-1.037	1.098	1.491	-0.789	0.983	1.117	1.126	1.346
		wheat	-1.040	-0.324	0.569	-0.960	-0.595	0.968	0.725	-0.288	-0.549	-0.401
D_60_	sole cropping	soybean	0.571	-0.950	-1.289	0.169	0.213	-1.837	-1.141	-0.794	-0.476	-0.544
		wheat	-1.172	-1.307	0.179	-1.102	-0.416	-0.464	-1.028	-1.451	-1.248	-1.186
	intercropping	soybean	0.837	0.806	-1.198	0.663	1.950	-1.164	0.291	0.515	0.313	0.455
		wheat	-1.121	-0.440	0.248	-1.002	-0.261	0.551	0.178	-0.520	-0.947	-1.007

^a^ “Z module” data is standardized from the raw module data.

D_20_, D_26_, D_32_ and D_60_ display 20, 26, 32 and 60 plants pot^-1^, respectively.

**Table 2:** Standardized data for population modules in soybean/wheat sole cropping and intercropping populations at low nutrient level and different planting densities.

Planting density	Planting mode	Crop type	^a^ (Z Leaf area)	^a^ (Z Leaf dry weight)	^a^ (Z Stem diameter)	^a^ (Z Stem dry weight)	^a^ (Z Plant height)	^a^ (Z Root tip number)	^a^ (Z Total root length)	^a^ (Z Root surface area)	^a^ (Z Root volume)	^a^ (Z Root dry weight)
D_20_	sole cropping	soybean	1.065	0.500	-0.716	0.872	-0.303	-0.489	-0.907	-0.103	0.538	0.027
		wheat	-0.858	-1.008	1.097	-0.918	-0.704	0.778	-0.796	-0.787	-1.017	-0.936
	intercropping	soybean	1.259	1.738	-0.670	1.365	1.616	-0.138	1.368	1.848	1.819	1.917
		wheat	-0.810	-0.120	1.395	-0.843	-0.336	1.649	1.142	0.611	-0.109	0.169
D_26_	sole cropping	soybean	1.138	0.798	-0.601	1.140	-0.478	-0.458	-0.822)	0.021	0.911	0.134
		wheat	-0.807	-0.870	1.579	-0.843	-1.028	1.077	-0.689	-0.665	-0.699	-0.686
	intercropping	soybean	1.307	1.942	-0.578	1.700	1.524	0.057	1.598	2.121	2.166	2.202
		wheat	-0.790	-0.003	1.602	-0.776	-0.487	1.923	1.355	0.704	0.165	0.348
D_32_	sole cropping	soybean	0.848	-0.491	-1.014	0.855	-0.146	-1.163	-0.976	-0.484	-0.078	-0.294
		wheat	-1.030	-1.140	0.661	-0.976	-0.592	0.193	-0.948	-1.139	-1.041	-1.007
	intercropping	soybean	1.063	1.177	-0.968	1.173	1.808	-0.647	1.052	1.279	1.245	1.417
		wheat	-1.002	-0.265	0.707	-0.893	-0.295	1.074	0.836	-0.195	-0.492	-0.330
D_60_	sole cropping	soybean	0.603	-0.885	-1.220	0.228	0.266	-1.227	-1.125	-0.716	-0.368	-0.472
		wheat	-1.139	-1.285	0.294	-1.043	-0.331	-0.335	-1.009	-1.361	-1.201	-1.114
	intercropping	soybean	0.862	0.857	-1.152	0.721	2.042	-1.029	0.342	90.623	0.404	0.490
		wheat	-1.103	-0.396	0.363	-0.951	-0.178	0.646	0.255	-0.457	-0.916	-0.972

^a^ “Z module” data is standardized from the raw module data.

D_20_, D_26_, D_32_ and D_60_ display 20, 26, 32 and 60 plants pot^-1^, respectively.

**Table 3:** Total variance interpretation and component matrix of population modules in soybean/wheat sole cropping and intercropping populations at different nutrient levels and planting densities.

**Modules**	Components		Initial eigenvalues			Extraction sums of squared loadings			^a^ U1	^b^ U2
	A1	A2	Total	% of Variance	Cumulative %	Total	% of Variance	Cumulative %		
Leaf area	0.889	-0.389	6.783	67.827	67.827	6.783	67.827	67.827	0.341	-0.245
**Leaf dry weight**	0.944	0.232	2.522	25.224	93.051	2.522	25.224	93.051	0.362	0.146
**Stem diameter**	-0.700	0.677	0.502	5.022	98.073				-0.269	0.426
**Stem dry weight**	0.931	-0.294	0.083	0.827	98.900				0.357	-0.185
**Plant height**	0.833	-0.045	0.058	0.582	99.481				0.320	-0.028
**Root tip number**	-0.436	0.883	0.029	0.287	99.768				-0.167	0.556
**Total root length**	0.548	0.792	0.012	0.123	99.891				0.210	0.499
**Root surface area**	0.863	0.498	0.005	0.050	99.942				0.331	0.314
**Root volume**	0.966	0.148	0.004	0.039	99.980				0.371	0.093
**Root dry weight**	0.939	0.310	0.002	0.020	100.000				0.361	0.195

^a^ U1 = A1/SQRT (6.783).

^b^ U2 = A2/SQRT (2.522).

**Table 4:** The PCA of the growth status of soybean/wheat population in their sole cropping and intercropping populations at different nutrient levels and planting densities.

Nutrient level	Planting density	Planting mode	Crop type	^a^ PC1	^b^ PC2	^c^ PCA scores	Nutrient level	Planting density	Planting mode	Crop type	^a^ PC1	^b^ PC2	^c^ PCA scores
½-strength Hoagland	D_20_	sole cropping	soybean	0.565	-1.759	-0.065ij	½-strength Hoagland	D_32_	sole cropping	soybean	-0.001	-2.466	-0.669k
			wheat	-2.994	0.062	-2.166no				wheat	-2.977	-0.636	-2.342o
		intercropping	soybean	4.137	0.841	3.244c			intercropping	soybean	3.489	0.005	2.544e
			wheat	-1.273	2.325	-0.298j				wheat	-1.611	1.324	-0.816kl
¼-strength Hoagland		sole cropping	soybean	1.017	-1.347	0.376gh	¼-strength Hoagland		sole cropping	soybean	0.337	-2.216	-0.355j
			wheat	-2.779	0.231	-1.963n				wheat	-2.838	-0.452	-2.191no
		intercropping	soybean	4.532	1.091	3.600b			intercropping	soybean	3.772	0.191	2.801d
			wheat	-0.916	2.642	0.049i				wheat	-1.411	1.524	-0.615k
½-strength Hoagland	D_26_	sole cropping	soybean	0.845	-1.496	0.210h	½-strength Hoagland	D_60_	sole cropping	soybean	-0.243	-2.855	-0.951l
			wheat	-3.013	0.451	-2.074no				wheat	-2.957	-1.185	-2.477p
		intercropping	soybean	4.477	1.103	3.562bc			intercropping	soybean	2.466	-0.997	1.527f
			wheat	-1.232	2.674	-0.173ij				wheat	-1.992	0.456	-1.328m
¼-strength Hoagland		sole cropping	soybean	1.389	-1.163	0.697g	¼-strength Hoagland		sole cropping	soybean	-0.195	-2.441	-0.804kl
			wheat	-2.696	0.776	-1.755ij				wheat	-2.865	-1.027	-2.367o
		intercropping	soybean	5.025	1.486	4.066a			intercropping	soybean	2.601	-0.840	1.669f
			wheat	-0.751	3.082	0.288gh				wheat	-1.910	0.616	-1.225m

^a^ PC1 = 0.341 × Z Leaf area + 0.362 × Z Leaf dry weight—0.269 × Z Stem diameter + 0.357 × Z Stem dry weight + 0.320 × Z Plant height—0.167 × Z Root tip number + 0.210 × Z Total root length + 0.331 × Z Root surface area + 0.371 × Z Root volume +0.361 × Z Root dry weight.

^b^ PC2 = - 0.245 × Z Leaf area + 0.146 × Z Leaf dry weight + 0.426 × Z Stem diameter—0.185 × Z Stem dry weight—0.028 × Z Plant height + 0.556 × Z Root tip number + 0.499 × Z Total root length + 0.314 × Z Root surface area + 0.093 × Z Root volume +0.195 × Z Root dry weight.

^c^ PCA scores = (67.827 × PC1 + 25.224 × PC2)/93.051.

PCA scores shown in Table 4 indicate the growth status of soybean and wheat populations in both sole cropping and intercropping populations; higher PCA scores indicate a better growth status. If the PCA scores of the soybean and wheat populations in their intercropping populations were higher than those in their sole cropping populations, positive interaction between the two populations in their intercropping populations was established. The strength of the positive interaction between both populations are shown using the PCA scores in the intercropping and sole cropping populations, i.e., greater changes in the PCA scores in both the intercropping and sole cropping populations indicated a stronger positive interaction. Our data for both nutrient levels indicated that positive interaction between soybean and wheat populations was established. This positive interaction was dependent on population density, and it was recorded as being strongest at D_26_, followed by D_20_, D_32_ and D_60_, in descending order. Furthermore, the effects of population density on the positive interaction under ¼-strength Hoagland treatment were more significant than those under ½-strength Hoagland treatment. Our data further indicated that D_26_ was the appropriate population density for positive interaction between both populations. In addition, as the population densities increased, the positive interaction between populations decreased. When the population density was constant, the positive interaction under ¼-strength Hoagland treatment was stronger than that under the ½-strength Hoagland treatment, indicating that low nutrient condition promoted positive interaction between populations.

## Discussion

Soybean/wheat intercropping populations are typical examples of mutually beneficial populations, and the link to maintain this mutually beneficial relationship is the positive interaction between populations. However, information relating to how nutrient levels and population density affect this positive interaction is currently lacking. A comparison between sole cropping and intercropping in this study verified that soybean and wheat populations are mutually beneficial when intercropped (Figs 1–3). This finding is consistent with previously published conclusions [26–29]. Furthermore, for a specific nutrient level (½- or ¼-strength Hoagland solution), all aboveground (except plant height) and belowground modules of soybean/wheat intercropping populations initially increased before decreasing as population density increased. These modules attained their maxima with a population density of D_26_ (Figs 1 and 2). However, plant height initially decreased before increasing as population density increased, reaching its minimum with a population density of D_26_ (Fig 1E). Thus, as population density increased, the positive interaction changed from being strong to being weak (Table 4). The change in interaction recorded here may be due to the following reasons. For a given nutrient level, D_20_ and D_26_ soybean/wheat intercropping populations had a larger growth space and more nutrient resources. Under these conditions, competition between the species was minimal and the positive interaction between the species resulted in them cooperatively utilizing resources (Table 4), resulting in the population modules recording good growth [30]. In addition, resources available for soybean and wheat plant individuals decreased as the population density increased, resulting in weak plant development and low plant height. Moreover, when the planting density of the soybean/wheat intercropping populations increased to D_32_ and D_60_, the environmental conditions became worse. For example, growth spaces between plants decreased and available nutrient resources also decreased. At this time, cooperation among the organisms in the intercropping populations weakened and population module growth slowed down, resulting in a change in the relationship between the intercropping populations from cooperation to competition (Table 4). The increase in plant height of the intercropping populations may be due to competition for light radiation resources and the preferential allocation of nutrients. These results were similar to those from previous investigations [31, 32]. However, due to the limitation of nutrient resources, it was not possible to meet the needs of simultaneous growth of other modules (such as stems) (Fig 1C). These phenomena indicated that an optimal population density exists in the soybean/wheat intercropping populations. An increase in planting density beyond the optimal population density weakened the positive interaction of both intercropping populations, thus forcing them to adjust their modules in response to the change in population density. Ultimately, this affected the positive interaction and resource utilization efficiency of the intercropping populations. Furthermore, our results indicated that, for a given population density, module growth observed in soybean/wheat intercropping populations under the ¼-strength Hoagland treatment were stronger than that under the ½-strength Hoagland treatment. This finding may be related to the relatively scarce nutrient resources that induced the production of a stress-growth response in the root modules to obtain more nutrients (Fig 3) to meet the growth requirements of the root modules (especially root length and root surface area) [33–36]. According to the correlation between the growths of belowground and aboveground modules, the expansion advantage of underground modules promoted the growth of the aboveground modules. Thus, both the belowground and aboveground modules under the ¼-strength Hoagland treatment were promoted in comparison to those under the ½-strength Hoagland treatment (Figs 1–3 and Table 4). However, it is foreseeable that under the condition of relative nutrient scarcity, the positive interaction resulting from the stress-growth response of population modules will be difficult to sustain.

Although this was a preliminary study, our findings indicated that, compared to natural populations, several constraints (such as nutrient level and planting density) affect the positive interaction between populations in soybean/wheat intercropping populations under agricultural conditions. The main limitation of this study was the analysis of ecological phenomena related to the effects of population density and nutrient levels on the positive interaction between artificial soybean/wheat intercropping populations based on the results of population modules. Accordingly, further assessments should be based on micro-ecological physiological processes related to changes in population modules, such as nutrient metabolism processes in the root module.

## Conclusions

(1) For the two nutrient levels (½- or ¼-strength Hoagland solution treatments) investigated in this study, soybean/wheat intercropping population modules initially increased before decreasing as population density increased. However, the positive interaction initially strengthened before becoming weak, reaching its maximum at D_26_. (2) Under the same planting density, ¼-strength Hoagland solution treatments promoted the growth of intercropping population modules and increased the positive interaction compared to ½-strength Hoagland solution treatments. (3) In these mutually beneficial soybean/wheat intercropping populations, the intensity of positive interaction between both crops was mediated by environmental nutrient level and population density. This phenomenon needs to be addressed when constructing intercropping populations.
